# Anti-malarial treatment outcomes in Ethiopia: a systematic review and meta-analysis

**DOI:** 10.1186/s12936-017-1922-9

**Published:** 2017-07-03

**Authors:** Eyob Alemayehu Gebreyohannes, Akshaya Srikanth Bhagavathula, Mohammed Assen Seid, Henok Getachew Tegegn

**Affiliations:** Department of Clinical Pharmacy, School of Pharmacy, University of Gondar-College of Medicine and Health Sciences, Gondar, Ethiopia

**Keywords:** Malaria, *Plasmodium falciparum*, *Plasmodium vivax*, Treatment, Efficacy, Safety, Adverse drug reactions, Artemether–lumefantrine, Chloroquine, Ethiopia

## Abstract

**Background:**

Ethiopia is among countries with a high malaria burden. There are several studies that assessed the efficacy of anti-malarial agents in the country and this systematic review and meta-analysis was performed to obtain stronger evidence on treatment outcomes of malaria from the existing literature in Ethiopia.

**Methods:**

A systematic literature search using the preferred reporting items for systematic review and meta-analysis (PRISMA) statement was conducted on studies from Pubmed, Google Scholar, and ScienceDirect databases to identify published and unpublished literature. Comprehensive meta-analysis software was used to perform all meta-analyses. The Cochrane *Q* and the *I* ^*2*^ were used to evaluate heterogeneity of studies. Random effects model was used to combine studies showing heterogeneity of Cochrane *Q* p < 0.10 and *I* ^*2*^ > 50.

**Results:**

Twenty-one studies were included in the final analysis with a total number of 3123 study participants. Treatment outcomes were assessed clinically and parasitologically using World Health Organization guidelines. Adequate clinical and parasitological response was used to assess treatment success at the 28th day. Overall, a significant high treatment success of 92.9% (95% CI 89.1–96.6), p < 0.001, *I* ^*2*^ = 98.39% was noticed. However, treatment success was higher in falciparum malaria patients treated with artemether–lumefantrine than chloroquine for *Plasmodium vivax* patients [98.1% (97.0–99.2), p < 0.001, *I* ^*2*^ = 72.55 vs 94.7% (92.6–96.2), p < 0.001, *I* ^*2*^ = 53.62%]. Seven studies reported the adverse drug reactions to anti-malarial treatment; of 822 participants, 344 of them were exposed to adverse drug reactions with a pooled event rate of 39.8% (14.1–65.5), *p* = 0.002.

**Conclusions:**

On the basis of this review, anti-malarial treatment success was high (92.9%) and standard regimens showed good efficacy against *Plasmodium falciparum* (98.1%) and *P. vivax* (94.7%) infections in Ethiopia, but associated with high rates of adverse drug reactions (ADRs). However, these ADRs were not serious enough to discontinue anti-malarial treatment. The results of this study suggest that the current anti-malarial medications are effective and safe; however, greater priority should be placed on the discovery of new anti-malarial drugs to achieve successful outcomes as resistance seems inevitable since cases of anti-malarial drug resistance have been reported from other areas of the world.

**Electronic supplementary material:**

The online version of this article (doi:10.1186/s12936-017-1922-9) contains supplementary material, which is available to authorized users.

## Background

Malaria is a vector-borne life-threatening disease caused by *Plasmodium* species that are transmitted to people through the bites of infected female *Anopheles* mosquitoes. It is characterized by fever, headache, chills, and vomiting and if not treated can progress to severe illness, often leading to death [1]. In 2015, an estimated 212 million cases and 429,000 deaths of malaria occurred globally [2]. Of these, around 90% of malaria cases and 92% of malaria deaths occurred in sub-Saharan Africa (SSA) [1, 2]. Ethiopia is also among countries with a high malaria burden. In 2015, an estimated 2.8 million cases and 4900 deaths due to malaria occurred in the country [2, 3].

Five *Plasmodium* species are responsible for malaria (*Plasmodium falciparum*, *Plasmodium vivax*, *Plasmodium malariae*, *Plasmodium ovale*, and *Plasmodium knowlesi*), but *P. falciparum* and *P. vivax* account for the largest threat [1–4]. *Plasmodium falciparum* is the most common (64%) cause of malaria in Ethiopia while *P. vivax* accounts for the remaining cases (34%) [3]. *Plasmodium falciparum* causes the most severe form of malaria, however, contrary to popular belief, *P. vivax* can also cause severe malaria and even death [5] and Ethiopia is home to the second highest number of cases [12% (n ~1020)] and mortality [12% (n ~372)] due to *P. vivax* (next only to India) in the world [2]. Malaria due to *P. vivax* and *P. ovale* are also characterized by relapses [6].

Reduced efficacy of chloroquine (CQ) [7, 8] has forced a change in the selection of anti-malarials in the management of falciparum malaria. Since 2004, Ethiopia has adopted artemether–lumefantrine (AL) and CQ as first-line treatment for infection with *P. falciparum* and *P. vivax*, respectively. In cases of treatment failure of *P. falciparum*, quinine (QN) is the treatment of choice and in cases of severe malaria, artemether, artesunate, or QN can be used [3, 9]. Globally, artemisinin resistance in *P. falciparum* has emerged, especially in Southeast Asia, slowing therapeutic response and increased rates of treatment failures [10, 11]. Similarly, artemisinin resistance has been reported from Africa although there is no evidence that it has taken hold currently [12]. Many studies assessing the efficacy of anti-malarial agents against *P. falciparum* and *P. vivax* have been published. However, no systematic review on this topic was identified from Ethiopia. Hence, this systematic review and meta-analysis was performed to obtain stronger evidence on treatment outcomes of malaria from the existing literature in Ethiopia.

## Methods

### Search strategies

The Cochrane guidelines to conduct the meta-analysis following the preferred reporting items for systematic review and meta-analysis (PRISMA) statement [13] was used to conduct a computerized systematic search of the PubMed, Google Scholar, and Science Direct databases. Prospective observational and interventional (randomized and single-arm) studies were included in the review using the following MeSH terms: malaria AND ethiopia AND (treatment OR management) AND (artemether–lumefantrine OR artesunate OR chloroquine OR mefloquine OR primaquine OR pyrimethamine) OR resistance. Only studies conducted in Ethiopia were included in the study. In addition to published researches, unpublished postgraduate thesis reports were also assessed to be included in the study. Publication dates were not used as inclusion or exclusion criteria and research papers published before 30 January, 2017 were included. Only those articles written in English language were considered for this review.

### Inclusion criteria

Papers fulfilling the following criteria were included in the study: studies presented as original articles; studies that examined malaria treatment outcomes; studies conducted in Ethiopia; studies written in English.

### Exclusion criteria

The following papers were excluded from the study: studies that used CQ for the treatment of falciparum malaria; studies that assessed treatment outcomes at times other than 28 days; studies where full articles were no longer available online (Additional file 1).

### Review process

**Fig. 1:**
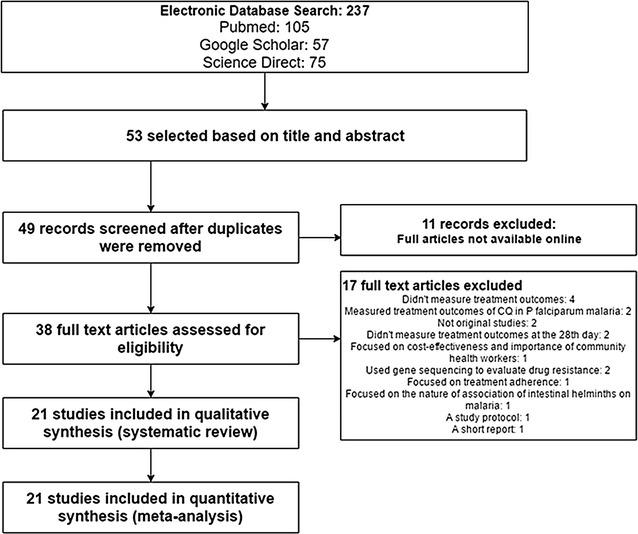
Flow diagram showing the number of articles identified in the systematic review on anti-malarial treatment outcomes in Ethiopia

All of the research articles that were identified from searches of the electronic databases were imported into the ENDNOTE software version X5 (Thomson Reuters, USA) and duplicates were removed. Before data extraction had begun, full-length articles of the selected studies were read to confirm for fulfilling the inclusion criteria. Three reviewers (EAG, MAS, HGT) independently screened the titles and abstracts to identify potentially eligible studies. Then data were extracted from full-length articles that fulfilled the inclusion criteria (Fig. 1). Discrepancies were resolved by mutual consent after discussion and independent review from the fourth researcher (ASB).

### Data extraction

Data on the types of study design (observational vs interventional), year the studies were conducted, length of study, and geographic location of the study area was first extracted. Participants’ age ranges, and gender of study participants were then extracted. Finally, data regarding the types of anti-malarial treatments, treatment outcome measures (including treatment success rates, treatment failure rates, and case fatality rates), treatment duration, and adverse drug reactions (ADRs) were extracted to be included in the systematic review and meta-analysis.

### Quality assessment and sensitivity analysis

Two reviewers (EAG and MAS) independently assessed methodological quality of studies using modified Jadad scale for randomized controlled trials (RCTs) [14] and Strengthening the Reporting of Observational Studies in Epidemiology (STROBE) statement for observational studies [15]. The scores for the modified Jadad scale can range from 0 to 8 (low to high quality). Scores of 4–8 represent good to excellent, whereas 0–3 represents low or poor quality. The observational studies were categorized as high quality (over 75% of the STROBE checklist) and low quality (under 75% of the STROBE checklist). To address the issue of heterogeneity of the studies, a sensitivity analysis was considered stratifying the studies into high quality and low quality.

### Statistical analysis

Comprehensive meta-analysis (CMA) software [16] was used to perform meta-analyses of proportions of success. The Cochrane *Q* and the *I* ^*2*^ were used to evaluate heterogeneity of studies. Random effects model was used to combine studies showing heterogeneity of Cochrane Q p < 0.10 and I^2^ > 50 [17]. A sub-group analysis was performed by comparing the treatment outcomes of patients with *P. falciparum* infection treated with AL and patients infected with *P. vivax* and treated with CQ. Rates of ADRs were also calculated for the different anti-malarial medications.

### Ethical consideration

This study was carried out in strict accordance with the recommendations in the PRISMA guidelines [13]. Since it is a systematic review and meta-analysis, ethics committee or institutional review board permission was not required.

## Results

An updated search of available literature identified 231 titles, of which two RCTs) [18, 19], 15 one-arm in vivo drug efficacy studies [20–34], and four prospective observational studies [35–38] were deemed eligible (Additional file 2). The 21 studies [18–38] included in this analysis came from individual treatment programmes across Ethiopia and reported treatment outcomes for a range of 69 [35] to 487 patients [26]. One unpublished Master’s thesis dissertation [36] was included and the rest were published in scientific journals [18–35, 37, 38]. Some 1868 patients with *P. falciparum* [21, 23–27, 30, 31, 34–38] and 1255 patients with *P. vivax* [18–20, 22, 28, 29, 32–35] were analysed with an age range between 6 months and 91 years.

The most commonly reported regimens were AL [18, 21, 23–25, 27, 30–32, 34–38] and CQ [18–20, 22, 28, 29, 32–35], and treatment outcomes were assessed using clinical and parasitological criteria according to four different World Health Organization (WHO) guidelines [39–42]. Adequate clinical and parasitological response (ACPR) was taken as treatment success. ACPR was defined as absence of parasitaemia by the end of treatment (day 28) irrespective of axillary temperature without previously meeting any of the criteria of early treatment failure or late clinical failure or late parasitological failure [40–42]. All of the studies included in the analysis assessed outcomes at 28 days.

### Malaria treatment success in Ethiopia

**Fig. 2:**
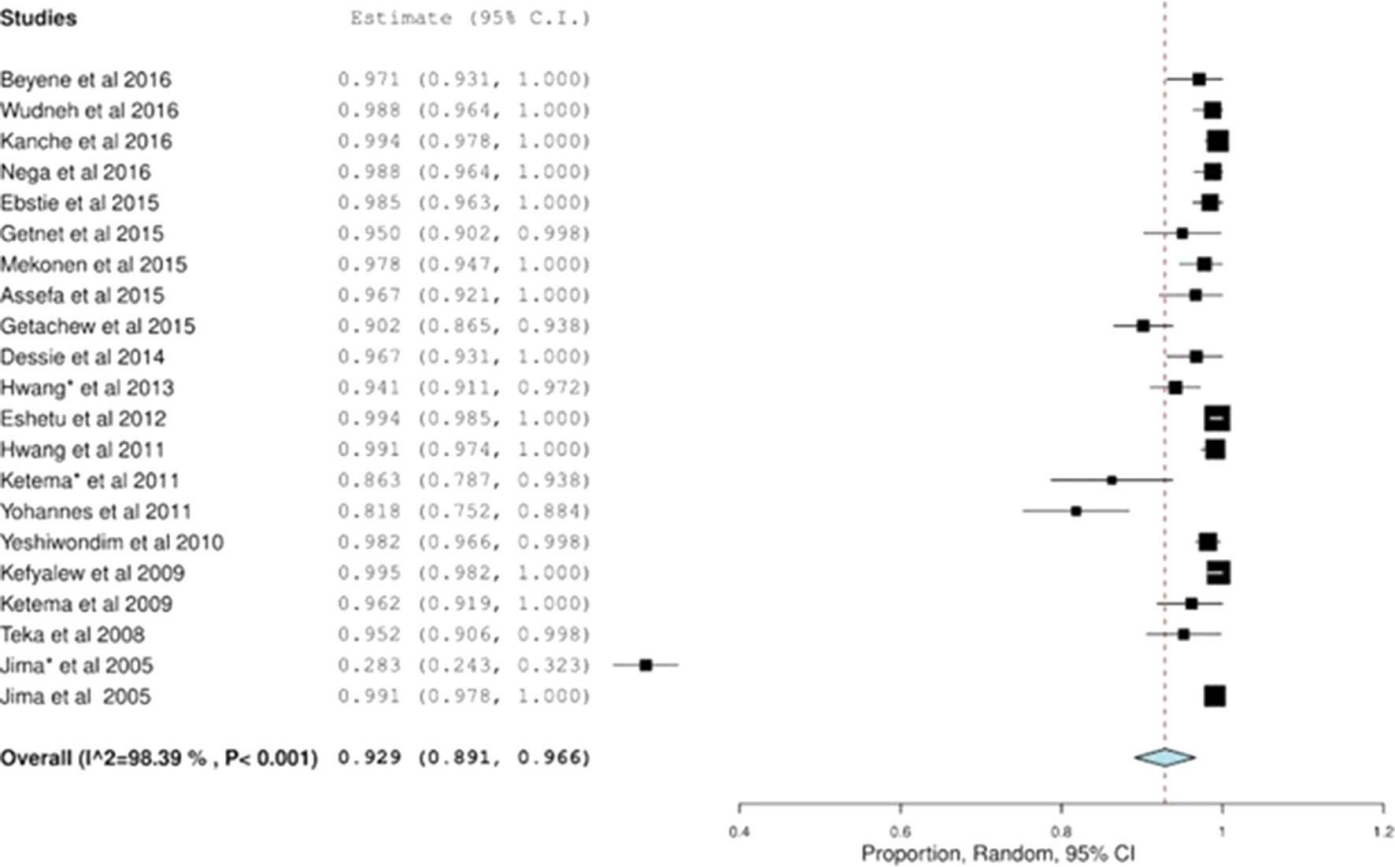
Overall treatment success

**Fig. 3:**
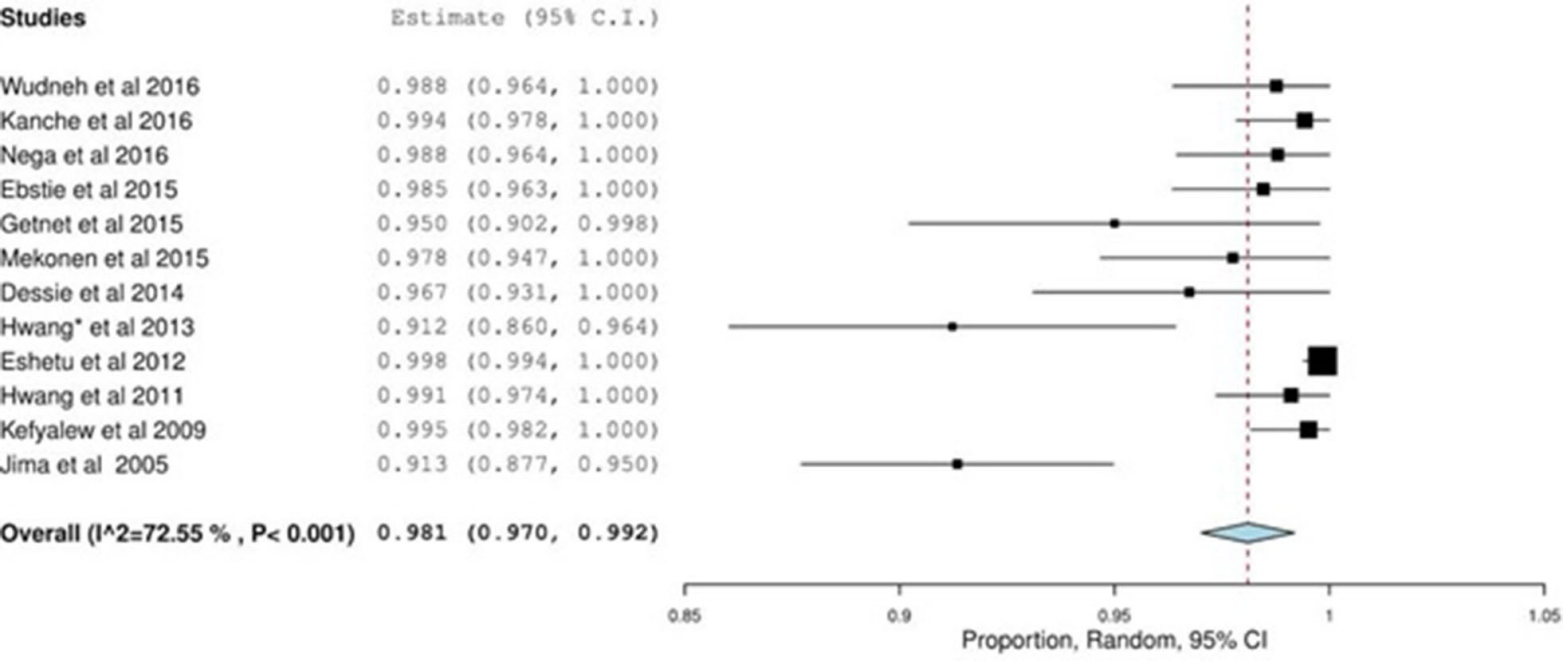
Treatment success with artemether–lumefantrine for *Plasmodium falciparum*

**Fig. 4:**
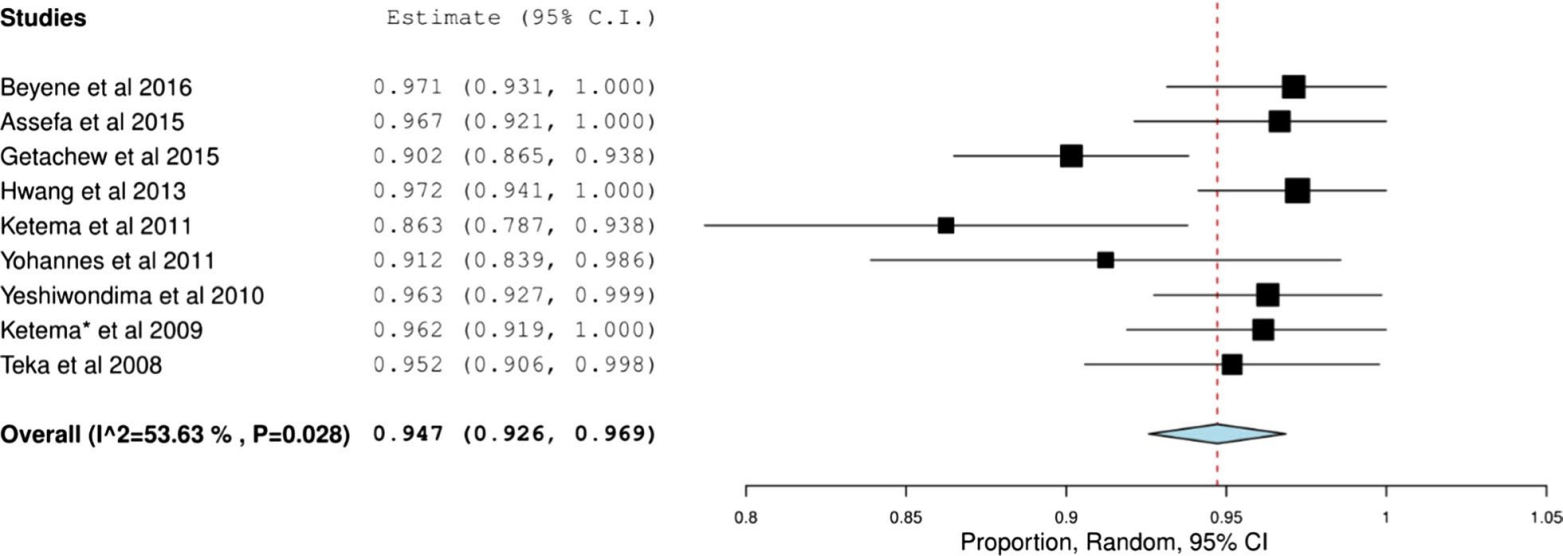
Treatment success with chloroquine for *Plasmodium vivax*

Twenty-one studies reported the treatment success in malaria patients with *P. falciparum* [21, 23–27, 30, 31, 34–38] and *P. vivax* [18–20, 22, 28, 29, 32–35] in Ethiopia. Only six studies used polymerase chain reaction (PCR) genotyping approach to differentiate recrudescence from new infections [18, 21, 23, 24, 30, 34]. For these six studies, the PCR-adjusted success and failure rates were used while PCR-unadjusted rates were used for the remaining studies. Overall, a significant high treatment success of 92.9% (95% CI 89.1–96.6), p < 0.001, *I* ^*2*^ = 98.39% (Fig. 2) was noticed. However, treatment success was higher in falciparum malaria patients treated with AL than CQ for *P. vivax* (98.1% (97.0–99.2), p < 0.001, *I* ^*2*^ = 72.55 vs 94.7% (92.6–96.2), p < 0.001, *I* ^*2*^ = 53.62%) (Figs. 3, 4).

### Malaria treatment failures in Ethiopia

Treatment failures were lower in patients with falciparum malaria treated with sulfadoxine–pyrimethamine (SP), AL with a pooled rate of 7.8% (2.1–13.6), *p* = 0.008. Nine studies reported the treatment failures in vivax malaria patients with a pooled rate of 7.9% (4.1–11.8), *p* < 0.001 (Table 1). Of all the study participants, only one patient died during the course of treatment [21].

**Table 1:** Pooled odds ratio of treatment failures and adverse drug reactions after exposure to artemether–lumefantrine and chloroquine among malaria patients in Ethiopia

	Number of studies	Treatment failure (n/N)	Events/participants (n/N)	Pooled event rate % (95% CI)	I^2^ (p value)
Overall treatment failures	21 studies (18–38)	*P. falciparum*: 373/1868; *P. vivax*: 114/1255	487/3123	8.0% (4.0–12.0)	98.42% (<0.001)
Treatment failure with *P. falciparum* patients	12 studies (21, 23–27, 30–31, 34–38)	AL = 21/1497	373/1868	7.8% (2.1–13.6)	99.07% (<0.001)
Treatment failures in *P. vivax* patients	9 studies (18–20, 22, 28–29, 32–35)	CQ = 66/897 CQ–PQ = 1/132 AL = 47/189	114/1255	7.9% (4.1–11.8)	88.19% (<0.001)
Overall ADRs	7 studies (13, 24, 27, 31, 35, 37, 38)	AL = 501/725 CQ = 75/189	344/822	39.8% (14.1–65.5)	99.46% (0.002)
ADRs to AL	6 studies (13, 24, 27, 31, 37, 38)	AL = 501/725	269/633	41.2% (10.1–72.4%)	99.5% (0.009)
ADRs to CQ	2 studies (18, 35)	CQ = 75/189	75/189	35.6% (5.7%–65.5%)	95.2% (0.020)

*AL* artemether–lumefantrine, *CQ* chloroquine, *CQ*–*PQ* chloroquine–primaquine, *MQ* mefloquine, *SP* sulfadoxine–pyrimethamine

### Adverse drug reactions during anti-malarial treatment in Ethiopia

Seven studies [13, 24, 27, 31, 35, 37, 38] reported ADRs to anti-malarial treatment; of 822 participants, 344 exposure to ADRs with a pooled event rate of 39.8% (14.1–65.5), *p* = 0.002. When examined the pooled events rates of anti-malarial drugs AL and CQ, AL has higher adverse events of 41.2% (10.1–72.4), p = 0.009 compared to CQ 35.6% (5.7–65.5), p = 0.020 (Table 1). Table 2 shows the most commonly reported ADRs.

**Table 2:** Most commonly reported adverse drug reactions

Drug	AL	CQ	Total
Sample size	725	189	1026
Weakness/fatigue	32	6	38
Abdominal discomfort/pain	43	13	56
Anorexia	20	2	22
Nausea/vomiting	19	19	38
Diarrhoea	11	7	18
Dizziness	14	4	18
Oral ulcer/inflammation	46	15	61
Cough/sore throat	51	7	58
Headache	59	4	63
Joint pain	12	0	12
Others	49	9	58
Total	356	86	442

### Quality assessment and sensitivity analysis

Seventeen interventional studies [18–34] were assessed using the modified Jadad scale [14] while the remaining four studies [35–38] were assessed with the STROBE statement [15]. Eleven interventional and two observational studies were judged to have high quality (Additional file 3).

Meta-analysis was stratified based on the quality of the studies (high and low quality) to investigate the overall treatment outcomes across the included studies. It revealed significant differences between the low-quality studies (86.1%, 95% CI 69.7–102.6, p = <0.001, I^2^ = 99.35%) [20, 26, 28, 30, 32, 33, 35, 38] and high quality studies (96.9%, 95% CI 95.4–98.3, p < 0.001; I^2^ = 81.7%) [18, 19, 21–25, 27, 29, 31, 36, 37]. Publication bias was also assessed using a funnel plot (Additional file 4).

## Discussion

This systematic review and meta-analysis was conducted mainly to estimate treatment outcomes of different anti-malarial medications used to treat both *P. falciparum* and *P. vivax* in Ethiopia. This systematic review identified 21 studies (from 2005 to 2016) that assessed the treatment outcomes of uncomplicated falciparum and vivax malaria in Ethiopia. These studies were conducted in the following provinces: Tigray, Amhara, Oromia, Benishangul-Gumuz, and the Southern Nations, Nationalities, and Peoples Region (SNNPR). Since poor efficacy of CQ in *P. falciparum* is well documented, AL replaced CQ as a treatment option for *P. falciparum* and CQ is no longer used in the management of falciparum malaria in Ethiopia. For this reason, studies that assessed treatment efficacy of CQ in *P. falciparum* were excluded from the analysis (Additional file 1). All of the studies used one or two of four WHO guidelines [39–42] to assess treatment outcomes of the study participants. Treatment outcomes were confirmed both clinically and parasitologically at 28 days for all studies.

This investigation suggested that a high proportion of treatment success of different anti-malarial drugs was seen in Ethiopia with an overall treatment success rate of 92.9%. However, the effectiveness varies between the species and the different anti-malarial drugs and most of the studies focused on AL and CQ for the management of *P. falciparum* and *P.* vivax infections, respectively. A pharmaceutical company-sponsored, pooled analysis of AL, which included seven studies, showed a 97.1% (95.2–98.3%) treatment success in patients with uncomplicated malaria [43]. This suggests that the results of treatment success with AL in uncomplicated malaria patients in Ethiopia are evenly distributed with other high malaria-endemic countries.

CQ has been found to have high treatment success rate (94.7%) for infection with *P. vivax*. Its superior efficacy over AL was demonstrated by two studies [18, 32] that compared the efficacy of both drugs against *P. vivax*. These studies showed that CQ has a better efficacy (90.9%) for the management of *P. vivax* than AL (75.1%). This superior efficacy of CQ over AL might be attributed to its longer elimination half-life with extended periods of post-treatment prophylaxis. Better efficacy of dihydroartemisinin–piperaquine, an artemisinin combination therapy with longer elimination half-life than AL, might support this justification [44]. Therefore, CQ is the preferred treatment option for the management of *P. vivax* infection in Ethiopia. However, despite the poorer efficacy of AL for *P. vivax* compared to CQ, it can be used in cases of mixed infections with *P. vivax* and *P. falciparum*. On the other hand, one study [19] compared the efficacy of CQ alone or in combination with PQ against *P. vivax*. Accordingly, the combination showed superior efficacy (99.2%) over CQ alone (96.3%). Likewise, a better parasitaemia clearance with combination of CQ and primaquine (PQ) was also seen in one study. Naing et al. [45] reported a higher rate of parasitaemia in patients treated with CQ alone when compared to patients who received PQ in combination with CQ (7.7 vs 4.9%). The standard treatment guideline for Ethiopia [9] recommends a 14-day therapy of PQ along with CQ as a first-line treatment in patients with *P. vivax* infections. However, despite lack of published reports, routine use of PQ suffers from availability issues.

In the current review, only one study participant died [21]. This lower rate of mortality is not surprising since the studies in this review included patients with uncomplicated malaria. However, high treatment failure rates of SP against *P. falciparum* were reported by Jima et al. [26]. Resistance of *P. falciparum* to SP has been well documented [46–49]. As a result, this combination is no longer included in the standard treatment guideline of the country [9]. A higher incidence of ADRs was noticed with AL than CQ (41.2 vs 35.6%). Detailed data of ADRs were absent from some studies, and these were not systematically recorded in most studies. Further, most studies reported ADRs that are similar to malaria symptoms, which might overlap. Nevertheless, more than one-third of adult patients on malaria treatment had experienced ADRs with AL and CQ.

Previous studies reported a higher incidence of ADRs to AL in adults with uncomplicated *P. falciparum* with serious adverse events rate of 1.4% [43]. This study recorded adverse event rates ranging from 72.8 to 86.1%. On the other hand, the current study identified that the adverse events to CQ in patients with *P. vivax* was more than 2.5 times (35.6 vs 13.3%) higher than the Naing et al. review within the same series [45]. Many of the adverse events reported are more frequent and consistent with other studies most of which are similar to well-recognized symptoms of malaria. Due to the similarities, under-reporting or over-reporting of ADRs might account for the observed differences. The higher rates of ADRs reported might be caused by an increased tendency to report symptoms during the course of anti-malarial treatment in Ethiopia. Apart from two infants who were unable to tolerate oral medications due to repeated vomiting [21], none of the studies reported serious ADRs that forced an individual to discontinue anti-malarial drug treatment. The infants were switched to intravenous drugs, but one of them died. However, there was no evidence whether the repeated vomiting in the two infants was due to the anti-malarial treatment.

This study has important implications for high malaria burden countries with high prevalence of *P. falciparum* and *P. vivax*. In view of WHO’s Global Technical Strategy for Malaria 2016–2030 to reduce global malaria incidence and mortality by at least 90% by 2030 [50], efficacy of artemisinin-combination anti-malarial drugs, particularly AL, showed promising results in treating patients with falciparum malaria and CQ for *P. vivax* in Ethiopia. However, resistance to anti-malarial drugs poses a major threat globally. On the basis of the results of this review, malaria treatment in Ethiopia is successful, but if the parasites develop resistance to these anti-malarial regimens, it is inevitable that treatment would be more difficult, unsuccessful and high rates of relapse could be due to multidrug-resistant malaria. Other implications are that as the previously used anti-malarial regimens were associated with higher rates of treatment failures in *P. falciparum* patients [7, 8], there is no guarantee how long the currently used anti-malarial drugs will remain effective. This supports calls for novel alternative anti-malarial drugs to treat malaria in the near future. Lastly, higher ADRs suggest the need for active surveillance and prospective follow-up are essential to determine the incidence of adverse events in malaria patients in Ethiopia.

This study has several strengths. A total of 21 studies were included that allowed results from a total of 3123 study participants. In addition to published research articles, unpublished researches were assessed for eligibility and one thesis dissertation was included in the analysis. The study included infection with both species, *P. falciparum* and *P. vivax*, and study outcomes were measured both clinically and parasitologically. The study assessed efficacy of commonly used anti-malarial drugs: AL, SP, CQ, and CQ–PQ. However, the current study is not without limitations. Only a few of the studies [18, 19, 32] evaluated the comparative efficacy of different anti-malarial medications and the current review relied mainly on one-arm studies, and only a few studies used PCR genotyping. Not all studies reported ADRs to anti-malarial drugs, which rendered the current study depend on fewer studies to assess the safety profiles of these drugs. None of the studies clearly reported how severity of ADRs was assessed and reporting on the severity of ADRs depended on their statement. Only studies written in English were included in the analysis as result studies written in other languages might be missed. Trials representing results from Ethiopia only were included, and a high heterogeneity was detected in the studies, which forced the random effect model to be applied reducing credibility ‎and increasing the imprecision of the results. Therefore, interpretation of the findings of this study should be in light of these limitations.

## Conclusions

On the basis of this review, anti-malarial treatment success was high (92.9%) and standard regimens showed good efficacy against *P. falciparum* (98.1%) and *P. vivax* (94.7%) infections in Ethiopia, but was associated with high rates of ADRs. However, the ADRs were not serious enough to discontinue anti-malarial treatment. The results of the current study suggest that current anti-malarial medications are effective and safe, however, greater priority should be placed on the discovery of new anti-malarial drugs to achieve successful outcomes as resistance seems inevitable since cases of anti-malarial drug resistance have been reported from other areas of the world.

## Additional files

**Additional file 1.** Excluded studies after review of full text articles.

**Additional file 2.** Overview of the included malaria studies conducted in Ethiopia from 2005 to 2016 (N = 3040).

**Additional file 3.** Quality assessment of included studies.

**Additional file 4.** Funnel plot: assessment of publication bias.
