# Supplementary material for: Anti-malarial treatment outcomes in Ethiopia: a systematic review and meta-analysis
Source: Malar J. 2017 Jul 3;16:269. doi: 10.1186/s12936-017-1922-9 (PMC5496337; doi:10.1186/s12936-017-1922-9)
Supplement: Supplementary file 1 — Additional file 1. Excluded studies after review of full text articles. [file 12936_2017_1922_MOESM1_ESM.docx]

# **Additional file 1: Excluded studies after review of full text articles**

|  | **Name** | **Title** | **Year** | **Journal** | **Reason** |
| --- | --- | --- | --- | --- | --- |
| 1 | Mavrogordato et al | A cluster of Plasmodium vivax malaria in an expedition group to Ethiopia: Prophylactic efficacy  Of atovaquone/proguanil on liver stages of P. Vivax | 2012 | Journal of Infection | Didn’t measure treatment outcomes |
| 2 | Degarege et al | Malaria and related outcomes in patients with intestinal helminths: a cross-sectional study | 2012 | BMC infectious diseases | Didn’t measure treatment outcomes |
| 3 | Scott et al | Mass testing and treatment for malaria in low transmission areas in Amhara region, Ethiopia | 2016 | BMC malaria | Didn’t measure treatment outcomes |
| 4 | Povinelli et al | Plasmodium vivax Malaria in Spite of Atovaquone/Proguanil (Malarone) Prophylaxis | 2003 | Journal of Travel Medicine | Didn’t measure treatment outcomes |
| 5 | Barnes et al | Impact of the large-scale deployment of artemether/lumefantrine  on the malaria disease burden in Africa: case studies of South  Africa, Zambia and Ethiopia | 2009 | BMC Malaria | Not an original study |
| 6 | Yohannes | Malaria Treatment in Ethiopia: Antimalarial Drug Efficacy Monitoring System and Use of Evidence for Policy | 2012 | Unpublished study: PHD Thesis Dissertation | Not an original study |
| 7 | Alene et al | Chloroquine resistance of Plasmodium falciparum malaria in Ethiopia and Eretria | 1996 | Tropical Medicine and International Health | Assessed efficacy of CQ for P falciparum malaria |
| 8 | Teklehaimanot | Chloroquine-Resistant Plasmodium Falciparum Malaria in Ethiopia | 1986 | The Lancet | Assessed efficacy of CQ for P falciparum malaria |
| 9 | Gidebo et al | Factors influencing malaria treatment and Patient adherence to antimalarial drugs in Southern Ethiopia | 2014 | Africa Journal of Nursing and Midwifery | Focused on malaria treatment practice and adherence |
| 10 | Reda | Improving efficiency, access to and quality of the rural Health Extension Programme in Tigray, Ethiopia: the case of malaria  diagnosis and treatment | 2012 | PhD thesis dissertation | Focused on cost-effectiveness and importance of community health workers |
| 11 | The IMPROVE Study Group | Improving the radical cure of vivax malaria (IMPROV): a study protocol for a multi-centre  randomised, placebo-controlled comparison of short and long course Primaquine regimens | 2015 | BMC infectious diseases | A study protocol |
| 12 | Tulu et al | Failure of chloroquine treatment for malaria in the highlands of Ethiopia | 1996 | Transactions of the Royal Society of Tropical Medicine and Hygiene | A short report |
| 13 | Lemma et al | Adherence to a six-dose regimen of artemether-lumefantrine  among uncomplicated Plasmodium falciparum patients in the Tigray Region, Ethiopia | 2011 | BMC Malaria | Focused on adherence |
| 14 | Kassa et al | Therapeutic efficacy of mefloquine and sulfadoxine/  pyrimethamine for the treatment of uncomplicated Plasmodium falciparum malaria in children, Metehara town, southeast Ethiopia | 2005 | Ethiop.J.Health Dev | Treatment outcomes not assessed at day 28 |
| 15 | Seboxa et al | Artemether-Lumefantrin (Coartem®) and Artesunate with  Sulfadoxine-Pyrimethamine Therapeutic Efficacy in the  Treatment of Uncomplicated Malaria at Gilgel Gibe II (GgII)  South-Western Ethiopia | 2010 | Ethiopian Medical Journal | Treatment outcomes not assessed at day 28 |
| 16 | Mekonnen et al | Return of chloroquine-sensitive Plasmodium falciparum parasites and emergence of chloroquine-resistant Plasmodium vivax in Ethiopia | 2014 | BMC Malaria | Used gene sequencing to evaluate drug resistance |
| 17 | Schunk et al | High prevalence of drug-resistance mutations in Plasmodium falciparum and Plasmodium vivax in southern Ethiopia | 2006 | BMC Malaria | Used gene sequencing to evaluate drug resistance |
