# Supplementary material for: Anti-malarial treatment outcomes in Ethiopia: a systematic review and meta-analysis
Source: Malar J. 2017 Jul 3;16:269. doi: 10.1186/s12936-017-1922-9 (PMC5496337; doi:10.1186/s12936-017-1922-9)
Supplement: Supplementary file 2 — Additional file 2. Overview of the included malaria studies conducted in Ethiopia from 2005 to 2016 (N = 3040). [file 12936_2017_1922_MOESM2_ESM.docx]

**Additional file 2: Overview of the included malaria studies conducted in Ethiopia from 2005-2016 (N=3040)**

| **Author** | **Journal** | **Year** | **Study design** | **Location** | **Study duration (Months)** | **Sample size** | **Age range** | **Type of Malaria** | **Treatment options** | **Outcomes** |
| --- | --- | --- | --- | --- | --- | --- | --- | --- | --- | --- |
| Jima et al | East African Medical Journal | 2005 | In vivo therapeutic efficacy and safety study | OR; AM; TG; SNNPR; GB | 3 | 487 | 6 mo to >15 years | *P falciparum* | SP | Success: 138; Failure: 349 |
| Teka et al | BMC Malaria | 2008 | In vivo therapeutic efficacy study | OR | 3 | 83 | 8 mo to 52 years | *P vivax* | CQ | Success: 79; Failure: 4 |
| Yeshiwondim et al | Acta Tropica | 2010 | prospective open-label randomized trial | OR | 8 | 277 | 4 to 65 years | *P vivax* | CQ; CQ-PQ | Success: 272; Failure: 5 |
| Beyene et al | PONE | 2016 | Prospective observational study | BG | 5 | 69 | 4 to 54 years | *P vivax* | CQ | Success: 67; Failure: 2 |
| Kefyalew et al | Parasite | 2009 | In vivo therapeutic efficacy study | SNNPR | 3 | 102 | <5 to >15 years | *P falciparum* | AL | Success: 102; Failure: 0 |
| Hwang et al* | PONE | 2013 | Randomized open label in vivo trial | OR | 4 | 222 | 1 to 70 years | *P vivax* | AL; CQ | Success: 184; Failure: 38 |
| Jima et al 2 | East African Medical Journal | 2005 | In vivo therapeutic efficacy and safety study | TG; OR | 3 | 213 | 6 mo to >15 years | *P falciparum* | AL | Success: 211; Failure: 2 |
| Wudneh et al | Therapeutics and Clinical Risk Management | 2016 | One-arm prospective in vivo trial | AM | 4 | 81 | 2 to 24 years | *P falciparum* | AL | Success: 80; Failure: 1 |
| Ebstie et al | BMC Malaria | 2015 | Prospective observational cohort | AM | 5 | 130 | 5 to 64 years | *P falciparum* | AL | Success: 128; Failure: 2 |
| Kanche et al | Journal of Biology, Agriculture and Healthcare | 2016 | One arm prospective observational study | SNNPR | 2 | 86 | 5 to >14 years | *P falciparum* | AL | Success: 86; Failure: 0 |
| Dessie | Unpublished AAU MSc Thesis | 2014 | One arm prospective observational study | BG | 3 | 92 | 3 to 60 years | *P falciparum* | AL | Success: 89; Failure: 3 |
| Ketema et al | BMC Malaria | 2009 | In vivo therapeutic efficacy study | OR | 4 | 78 | 9 mo to 45 years | *P vivax* | CQ | Success: 75; Failure: 3 |
| Hwang et al | BMC Malaria | 2011 | Single arm in vivo therapeutic efficacy trial | OR | 2 | 112 | <5 to >5 years | *P falciparum* | AL | Success: 111; Failure: 1 |
| Eshetu et al* | BMC Malaria | 2012 | Single arm open-label in vivo therapeutic efficacy trial | OR | 8 | 313 | 1 to 91 years | *P falciparum* | AL | Success: 312; Failure: 3 |
| Ketema et al | BMC Parasites and vectors | 2011 | In vivo therapeutic efficacy trial | SNNPR | 2 | 80 | 9 mo to 52 years | *P vivax* | CQ | Success: 69; Failure: 11 |
| Getnet et al* | BMC Malaria | 2015 | Single arm in vivo therapeutic efficacy trial | AM | 5 | 80 | <5 to >5 years | *P falciparum* | AL | Success: 74; Failure: 6 |
| Mekonen et al* | BMC Malaria | 2015 | In vivo therapeutic efficacy trial | OR | 4 | 89 | <5 to >5 years | *P falciparum* | AL | Success: 84; Failure: 5 |
| Assefa et al | BMC Malaria | 2015 | Single-arm in vivo therapeutic efficacy trial | SNNPR | 3 | 60 | 4 to 59 years | *P vivax* | CQ | Success: 58; Failure: 2 |
| Getachew et al* | BMC Malaria | 2015 | In vivo therapeutic efficacy trial | SNNPR | 44 | 288 | 8 mo to 65 years | *P vivax* | CQ | Success: 229; Failure: 25 |
| Yohannes et al | AM. J. Trop. Med. Hyg | 2011 | In vivo therapeutic efficacy trial | N/A | Not mentioned | 132 | <5 to >5 years | *P. vivax* | CQ; AL | Success: 108; Failure: 24 |
| Nega et al* | Plos One | 2016 | Single-arm in vivo therapeutic efficacy trial | OR | 4 | 83 | 1 to 49 years | *P. falciparum* | AL | Success: 82  Failure: 1 |

***PCR-adjusted; AL: Artemether-Lumefantrine; AM: Amhara, BG: Benishangul-Gumuz, CQ: Chloroquine; OR: N/A: Not available, Oromia, PQ: Primaquine; SP: Sulfadoxine-Pyrimethamine; SNNPR: South Nations Nationalities and Peoples Region, TG: Tigray**
