# Supplementary material for: Anti-malarial treatment outcomes in Ethiopia: a systematic review and meta-analysis
Source: Malar J. 2017 Jul 3;16:269. doi: 10.1186/s12936-017-1922-9 (PMC5496337; doi:10.1186/s12936-017-1922-9)
Supplement: Supplementary file 3 — Additional file 3. Quality assessment of included studies. [file 12936_2017_1922_MOESM3_ESM.docx]

# **Additional file 3: QUALITY ASSESSMENT OF INCLUDED STUDIES**

## **Observational studies**

|  | **Study** | **Quality Assessment Tool** | **Score (out of 22)** | **Score (percentage)** | **Quality** |
| --- | --- | --- | --- | --- | --- |
| 1 | Dessie et al 2014 | STROBE | 20 | 91 | High |
| 2 | Ebstie et al 2015 | STROBE | 17 | 85 | High |
| 3 | Beyene et al 2016 | STROBE | 16 | 72 | Low |
| 4 | Kanche et al 2016 | STROBE | 15 | 68 | Low |

STROBE: Strengthening the Reporting of Observational Studies in Epidemiology

## **Interventional studies including randomized controlled trials**

|  | **Study** | **Quality Assessment Tool** | **Score (out of 8)** | **Quality** |
| --- | --- | --- | --- | --- |
| 1 | Jima et al 2005 | Modified Jadad Scale | 3 | Low |
| 2 | Jimma et al* 2005 | Modified Jadad Scale | 4 | High |
| 3 | Teka et al 2008 | Modified Jadad Scale | 3 | Low |
| 4 | Kefyalew et al 2009 | Modified Jadad Scale | 4 | High |
| 5 | Ketema et al 2009 | Modified Jadad Scale | 3 | Low |
| 6 | Yeshiwondim et al 2010 | Modified Jadad Scale | 5 | High |
| 7 | Hwang et al 2011 | Modified Jadad Scale | 4 | High |
| 8 | Ketema et al 2011 | Modified Jadad Scale | 4 | High |
| 9 | Yohannes et al 2011 | Modified Jadad Scale | 3 | Low |
| 10 | Eshetu et al 2012 | Modified Jadad Scale | 4 | High |
| 11 | Hwang 2013 | Modified Jadad Scale | 6 | High |
| 12 | Assefa et al 2015 | Modified Jadad Scale | 3 | Low |
| 13 | Getnet et al 2015 | Modified Jadad Scale | 4 | High |
| 14 | Getachew et al 2015 | Modified Jadad Scale | 4 | High |
| 15 | Mekonen et al 2015 | Modified Jadad Scale | 3 | Low |
| 16 | Wudneh et al 2016 | Modified Jadad Scale | 4 | High |
| 17 | Nega et al 2016 | Modified Jadad Scale | 4 | High |
