# Supplementary material for: Anti-malarial treatment outcomes in Ethiopia: a systematic review and meta-analysis
Source: Malar J. 2017 Jul 3;16:269. doi: 10.1186/s12936-017-1922-9 (PMC5496337; doi:10.1186/s12936-017-1922-9)
Supplement: Supplementary file 4 — Additional file 4. Funnel plot: assessment of publication bias. [file 12936_2017_1922_MOESM4_ESM.docx]

**
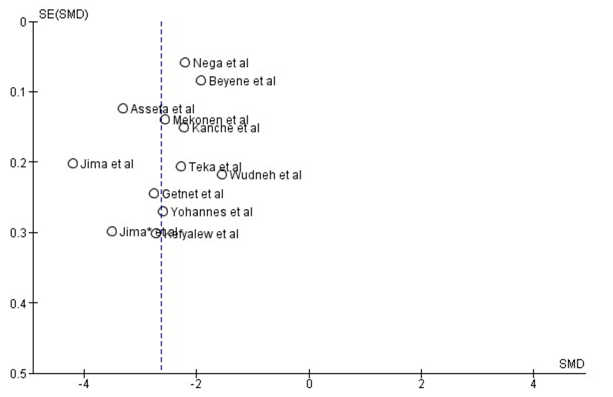
**

***SE: Standard Error; SMD: Standard Mean Difference***

**Additional file 4: Funnel plot: Assessment of publication bias**
